# Supplementary material for: Programming DNA Reaction Networks Using Allosteric DNA Hairpins
Source: Biomolecules. 2023 Mar 5;13(3):481. doi: 10.3390/biom13030481 (PMC10046357; doi:10.3390/biom13030481)
Supplement: Supplementary file 1 [file biomolecules-13-00481-s001.zip › biomolecules-2146282-supplementary.pdf]

Supporting Information: Programming DNA Reaction Networks using Allosteric DNA Hairpins

Table of Contents

1 Materials……………………………………………………………………….2

2 Methods ……………………………………………………………………….2

2.1 DNA assembly………………………………………………………….……2

2.2 Native Polyacrylamide Gel Electrophoresis (PAGE) …………………………….2

2.3 Fluorescence spectroscopy………………………………..……………..…..2

2.4 Mg^{2+}-ion-dependent DNAzyme dynamic assembly. ……………………...…..4

2.5 Hemin/G-quadruplex DNAzyme dynamic assembly..…………………..………6

2.6 Single-input Dual-output Module ……………………......………………...…10

2.7 Reversible regulatory DNA reaction networks……………….....……………12

3 DNA sequences….……………………………………………………….. 14

Figure S1 …………………………………………………………...................4

Figure S2 ………………………………………………………….................. 5

Figure S3………………………………………………………….................. 6

Figure S4…………………………………………………………...................7

Figure S5…………………………………………………………................... 8

Figure S6 …………………………………………………………...................9

Figure S7 ………………………………………………………….............. 10

Figure S8………………………………………………………….............. 11

Figure S9…………………………………………………………................12

Table S1…………………………………………………………................... 14

1 Materials

All DNA primers were purchased from Sangon Biotech. Co., Ltd. (Shanghai, China). Unmodified primers were purified by polyacrylamide gel electrophoresis (PAGE), and primers with a fluorophore or RNA base modification were purified by high-performance liquid chromatography (HPLC). All the DNA primers needed for the experiment were dissolved in ultra-pure water and quantified using a Nanodrop 2000 spectrophotometer. (Thermo Fisher Scientific Inc., Waltham, MA, USA). 2,2'-Azinobis (3-ethylbenzothiazoline-6-sulfonic Acid Ammonium Salt) [ABTS] was purchased from Tci Development Co., Ltd. (Shanghai, China). Hydrogen peroxide was purchased from Jiangxi Caoshanhu Disinfection Co., Ltd. (Jiangxi, China). Hemin and dimethyl sulfoxide were purchased from Sangon Biotech Co., Ltd. (Shanghai, China). Other chemicals were of reagent grade and were used without further purification.

2 Methods

2.1 DNA assembly

All DNA hairpins were annealed by using the same annealing procedure. DNA strands were annealed in a 1× TAE/Mg^{2+} buffer (40 mM Tris, 20 mM acetic acid, 100 mM NaCl, 1 mM EDTA·2Na, and 12.5 mM Mg (OAc) 2; pH 8.0). The solution was heated to 95°C for 10 min and then cooled to 25°C at 1°C per minute and preserved at 25°C for 1.5 h. The trigger strand was added to the annealed hairpins to realize the assembly of the DNAzyme. All DNA sequences required for the experiments were designed using the NUPACK. We use it to analyze free energy, simulate DNA strands binding, check base mismatches, and optimize the design sequence.

2.2 Native PAGE

First, 15 pmol hairpin was added to 30 μL 1× TAE/Mg^{2+} buffer, and then, 15 pmol trigger strand was added. After letting the reaction proceed for 4 h, 5 μL of 60% glycerol solution was added, and then, the system was analyzed on a 12% native polyacrylamide gel. The 12% native polyacrylamide gel was prepared and run on a BIO-RAD electrophoresis apparatus (BIO-RAD Co., California, USA) at a constant voltage of 80 V in 1× TAE/Mg^{2+} buffer for 2.5 h.

2.3 Fluorescence spectroscopy

In the first two modules, Mg^{2+}-ion-dependent DNAzyme dynamic assembly and hemin/G-quadruplex DNAzyme dynamic assembly. The fluorescence results were obtained using a TECAN Microplate Reader Spark 20M. (Tecan Trading Co., Grödig Australia). The fluorescence intensity was recorded per minute. The fluorescence analysis method for the Mg^{2+}-ion-dependent DNAzyme was as follows. First, 15 pmol hairpins H1, H2, H3 and the reporter strand and different concentrations of trigger strand T1 were added to 40 μL 1× TAE/Mg^{2+} buffer for 4 h, and then, the fluorescence of the FAM fluorophore between 500 and 600 nm was recorded at 25°C. The signal analysis method for the hemin/G-quadruplex DNAzyme was as follows. First, 40 pmol of the hairpins H4, H5, and H6; KCL (4 μL, 500 mM); and 0.8 μL Hemin (50 μM) and different concentrations of trigger strand T2 were reacted in 40

μL 1× TAE/Mg^{2+} buffer for 4 h; then, $ABTS^{2-}$ (16 μL, 2.5 mM), H_2O_2 (6.4 μL, 50 mM), and ultrapure water (17.6 μL) were added. The final measured volume was 80 μL. The absorbance of $ABTS^{•-}$ between 400 and 500 nm was recorded at 25°C. All fluorescence experiments were repeated three times to ensure reproducibility.

In the single-input dual-output module. DNAzyme 1 and DNAzyme 2 were analyzed as follows. First, 15 pmol hairpins H1, H2, and H3 and the reporter strand and 40 pmol of the hairpins H4, H5, and H6; KCL (4 μL, 500 mM); and 0.8 μL Hemin (50 μM) and 55 pmol trigger strand were added to 40 μL 1× TAE/Mg^{2+} buffer for 4 h, and two such solutions were prepared. One of them was selected and the fluorescence of FAM between 500 nm and 600 nm was recorded in one sample at 25℃. Choose another portion and add $ABTS^{2-}$ (16 μL, 2.5 mM), H_2O_2 (6.4 μL, 50 mM) and ultrapure water (17.6 μL). The final measured volume was 80 μL. At 25°C, the absorbance of $ABTS^{•-}$ at 420 nm was recorded.

In the the reversible regulatory DNA reaction network. DNAzyme 1 and DNAzyme 2 were analyzed as follows. First, fluorescence analysis method for the Mg^{2+}-ion-dependent DNAzyme was as follows. 15 pmol hairpins H1, H2, and H3 and the reporter strand and 40 pmol of the hairpins H4, H5, and H6; KCL (4 μL, 500 mM); and 0.8 μL Hemin (50 μM) and 55 pmol trigger strand S were added to 40 μL 1× TAE/Mg^{2+} buffer for 1.5 h, then the inhibitor C1 reaction was then added for 1.5 h, then the de-inhibitor C1* reaction was then added for 1.5 h, this cycle is repeated three times, the fluorescence of FAM between 500 nm and 600 nm was recorded in one sample at 25℃. The second, signal analysis method for the hemin/G-quadruplex DNAzyme was as follows. 15 pmol hairpins H1, H2, and H3 and the reporter strand and 40 pmol of the hairpins H4, H5, and H6; KCL (4 μL, 500 mM); and 0.8 μL Hemin (50 μM) and 55 pmol trigger strand S were added to 40 μL 1× TAE/Mg^{2+} buffer for 1.5 h, then the inhibitor C1 reaction was then added for 1.5 h, then the de-inhibitor C1* reaction was then added for 1.5 h, this cycle is repeated three times, at every point in time add $ABTS^{2-}$ (16 μL, 2.5 mM), H_2O_2 (6.4 μL, 50 mM) and ultrapure water (17.6 μL). The final measured volume was 80 μL. At 25°C, the absorbance of $ABTS^{•-}$ at 420 nm was recorded. Then the absorbance value of each time point of sample addition was taken to fit the curve.

2.4 The principle and verification of the Mg^{2+}-ion-dependent DNAzyme dynamic assembly module

In the Mg^{2+}-ion-dependent DNAzyme dynamic assembly module, fluorescence intensity control experiments were conducted to analyze the catalytic effects of different concentrations of catalyst T1 (Figure S1a and 1b). A series of catalyst T1 concentrations as 0 μM (0×), 0.075 μM (0.2×), 0.15 μM (0.4×), 0.225 μM (0.6×), 0.3 μM (0.8×), and 0.375 μM (1×) were introduced to assemble the Mg^{2+}-ion-dependent DNAzyme. As shown in Figure S1a, Time-dependent fluorescent intensity changes were implemented to verify the catalytic effects of different concentration of catalyst strand T1. As the catalyst T1 concentration increased, the fluorescent intensity increased correspondingly. After a certain time, the same fluorescence intensity was achieved by adding different concentrations of trigger strand T1. As shown in Figure S1b, fluorescence intensity at wavelength 500 nm to 600 nm was measured different concentrations of T reacting for 4 h. It can be seen from Figure S1b that 0.8× and 1× achieve the same fluorescence intensity. It shows that the catalytic effect is achieved. In order to further verify the catalytic effect. That is, the trigger strand T1 acts as a catalyst in the whole process.

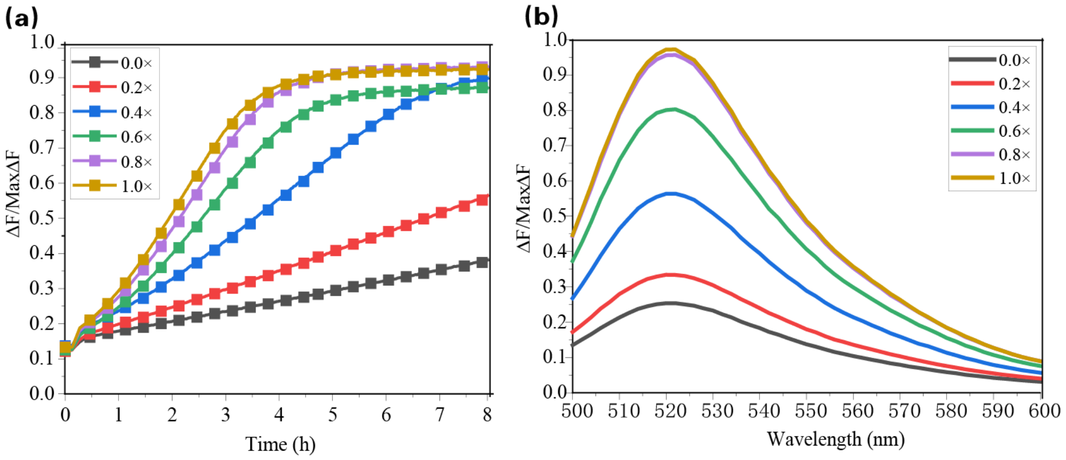

Figure S1. Analytical plot of the intensity of the T1 signal amplification of the trigger strand. (a) Time-dependent fluorescent intensity changes of different concentrations of catalyst strand T1 in the Mg^{2+}-ion-dependent DNAzyme dynamic assembly module. The different concentrations of T1 as 0 μM (0×), 0.075 μM (0.2×), 0.15 μM (0.4×), 0.225 μM (0.6×), 0.3 μM (0.8×), and 0.375 μM (1×). (b) Fluorescence intensity of different concentration strand T1 reaction for 4 hours at wavelength 500-600nm. Different concentrations strand T1 as 0 μM (0×), 0.075 μM (0.2×), 0.15 μM (0.4×), 0.225 μM (0.6×), 0.3 μM (0.8×), and 0.375 μM (1×) and substrate hairpin reactions were triggered at 25 ° C for 4 h.

To further verify the mechanism described in Figure S1, CHA hairpin H2 was transformed into fH2, respectively, in which the part of H2 of CHA hairpin that could replace T1 was replaced by a polythymidines (T) sequence. Thus, CHA-mediated autonomous H1, H2, H3 hybridization cannot lead to the regeneration of T1. As shown in Figure S2, the green bar represents the DNA reaction network with CHA mechanism, while the purple bar represents the DNA reaction network without CHA mechanism. The results show that the DNA reaction network with the assistance of CHA mechanism has basically reached the maximum value in 5 hours. However, the reaction rate of DNA reaction network without CHA mechanism has been increasing, so it can be seen that CHA mechanism accelerates the reaction rate to some extent. Therefore, our mechanism not only has the effect of signal amplification, but also has a certain improvement in the reaction rate.

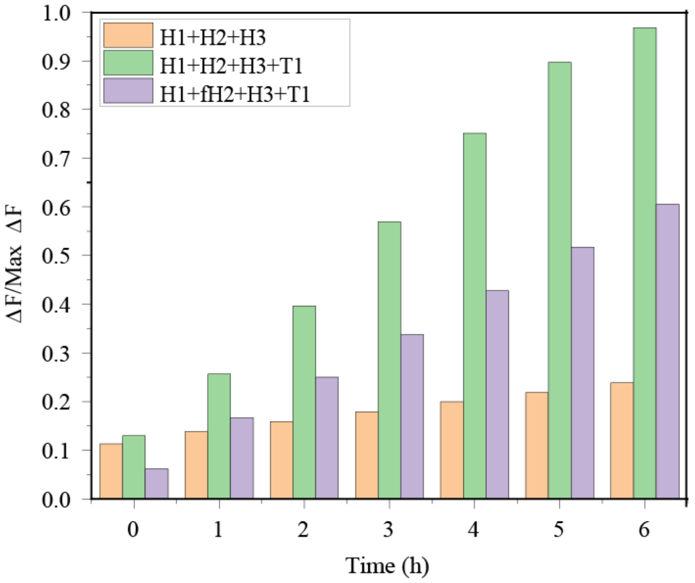

Figure S2. Bar graph of the catalytic effect of trigger strand T1, the time-dependent fluorescence changes (at λ=520 nm). T1 was used as the fluorescence curve of catalyst (green bar) and non-catalyst (purple bar), and the concentration of T1 was 0.6×. (green bar) H1+H2+H3+T1; (purple bar) H1+fH2+H3+T1; (orange bar) H1+H2+H3;

2.5 The principle and verification of the hemin/G-quadruplex DNAzyme dynamic assembly module

The conditional exploration of the hemin/G-quadruplex DNAzyme dynamic assembly module. First of all, the absorbance of the $ABTS^{\bullet-}$ changes were implemented to verify the appropriate concentration of H_2O_2. As shown in Figure S3, a series of H_2O_2 concentrations as 0.5 mM, 2 mM, 4.0 mM, 6.0 mM and 8.0 mM were introduced to assemble hemin/G-quadruplex DNAzyme. As the H_2O_2 concentration increased, the absorbance of the $ABTS^{\bullet-}$ increased correspondingly. However, the absorbance no longer rises after the H_2O_2 concentration increases to a certain level, instead of going down, so we choose 4.0 mM as the most appropriate concentration of H_2O_2 for our following experiments.

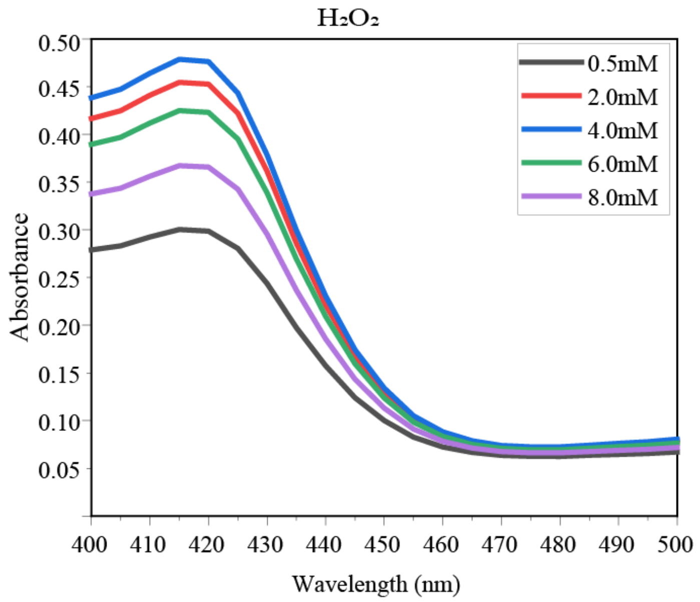

Figure S3. The absorbance of the $ABTS^{\bullet-}$ at different concentrations H_2O_2 as 0.5 mM, 2 mM, 4.0 mM, 6.0 mM and 8.0 mM.

At the same time, the absorbance of the $ABTS^{•−}$ changes were implemented to verify the appropriate concentration of Hemin. As shown in Figure S4, a series of Hemin concentrations as 0.0 μM, 0.3 μM, 0.6 μM, 1.0 μM and 1.5 μM were introduced to assemble hemin/G-quadruplex DNAzyme. As the Hemin concentration increased, the absorbance of the $ABTS^{•−}$ increased correspondingly. However, the absorbance no longer rises after the Hemin concentration increases to a certain level, so we choose 1.0 μM as the most appropriate concentration of Hemin for our following experiments.

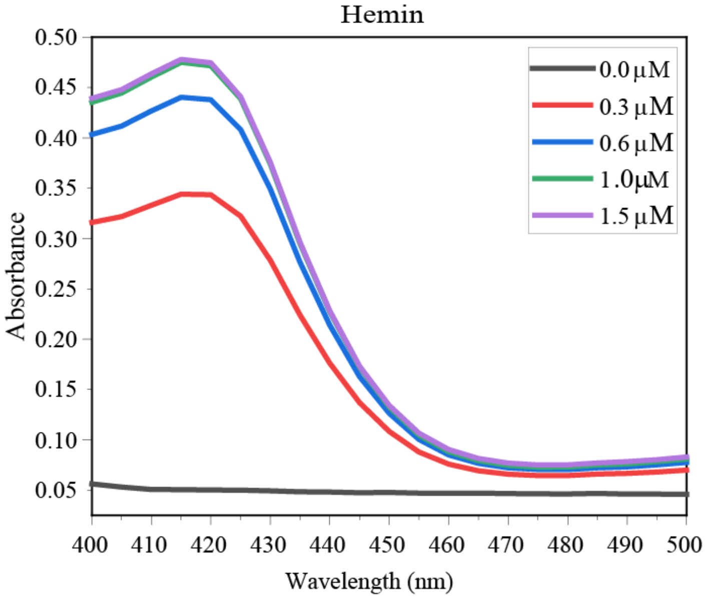

Figure S4. The absorbance of the $ABTS^{•−}$ at different concentrations Hemin as 0.0 μM, 0.3 μM, 0.6 μM, 1.0 μM and 1.5 μM.

In the hemin/G-quadruplex DNAzyme dynamic assembly module, the absorbance of the $ABTS^{\bullet-}$ control experiments were conducted to analyze the catalytic effects of different concentrations of catalyst T2 (Figure S5a and 5b). A series of catalyst T2 concentrations as 0 μM (0×), 0.2 μM (0.2×), 0.4 μM (0.4×), 0.6 μM (0.6×), 0.8 μM (0.8×), and 1 μM (1×) were introduced to assemble the hemin/G-quadruplex DNAzyme. As shown in Figure S5a, Time-dependent the absorbance of the $ABTS^{\bullet-}$ changes were implemented to verify the catalytic effects of different concentration of catalyst strand T2. As the catalyst T2 concentration increased, the absorbance of the $ABTS^{\bullet-}$ increased correspondingly. After a certain time, the same the absorbance of the $ABTS^{\bullet-}$ was achieved by adding different concentrations of trigger strand T2. As shown in Figure S5b, the absorbance of the resulting $ABTS^{\bullet-}$ at wavelength 400 to 500 was measured different concentrations of T2 reacting for four hours. It can be seen from Figure S5b that 0.8× and 1× achieve the same absorbance. It shows that the catalytic effect is achieved. In order to further verify the catalytic effect. That is, the trigger strand T2 acts as a catalyst in the whole process.

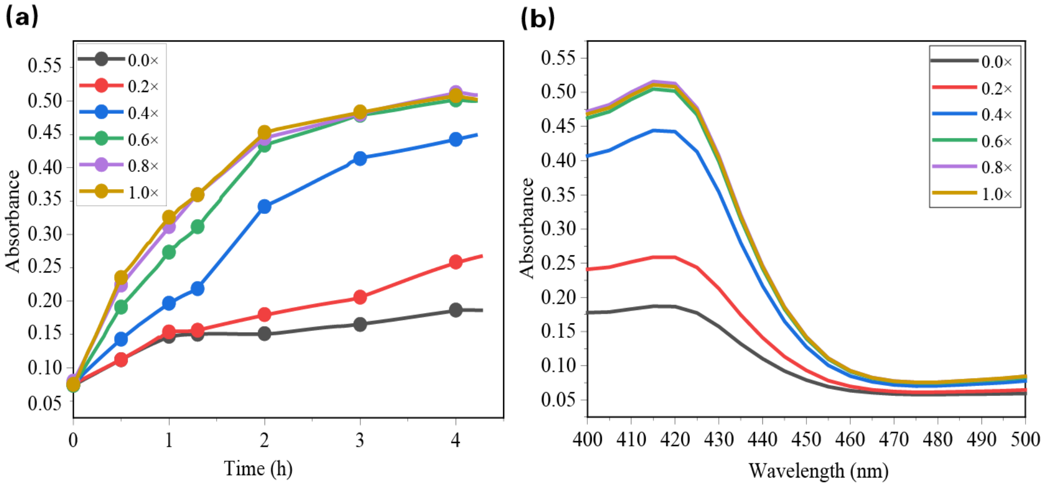

Figure S5. Analytical plot of the intensity of the T2 signal amplification of the trigger strand. (a) Time-dependent fluorescent intensity changes of different concentrations of catalyst strand T2 in the hemin/G-quadruplex DNAzyme dynamic assembly module. The different concentrations of T2 as 0 μM (0×), 0.2 μM (0.2×), 0.4 μM (0.4×), 0.6 μM (0.6×), 0.8 μM (0.8×), and 1 μM (1×). (b) Exploration and analysis of catalytic effect of trigger strand T2. (a) The absorbance of the $ABTS^{\bullet-}$ of different concentration strand T1 reaction for 4 hours at wavelength 500-600nm. Different concentrations strand T2 as 0 μM (0×), 0.2 μM (0.2×), 0.4 μM (0.4×), 0.6 μM (0.6×), 0.8 μM (0.8×), and 1 μM (1×) and substrate hairpin reactions were triggered at 25 ° C for 4 h.

To further verify the mechanism described in Figure S6, CHA hairpin H5 was transformed into fH5, respectively, in which the part of H5 of CHA hairpin that could replace T2 was replaced by a polythymidines (T) sequence. Thus, CHA-mediated autonomous H4, H5, H6 hybridization cannot lead to the regeneration of T2. As shown in Figure S6, the optimization effect of DNA reaction network with the assistance of CHA mechanism (trigger strand concentration is 0.6×). The green bar represents the DNA reaction network with CHA mechanism, while the purple bar represents the DNA reaction network without CHA mechanism. The results show that the DNA reaction network with the assistance of CHA mechanism has basically reached the maximum value in 5 hours. However, the reaction rate of DNA reaction network without CHA mechanism has been increasing, so it can be seen that CHA mechanism accelerates the reaction rate to some extent. Therefore, our mechanism not only has the effect of signal amplification, but also has a certain improvement in the reaction rate.

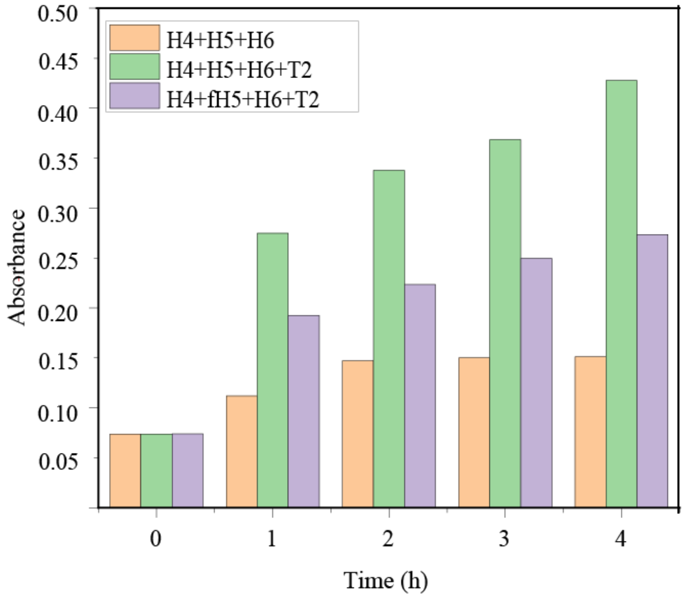

Figure S6. Bar graph of the catalytic effect of trigger strand T2, the time-dependent the absorbance of the $ABTS^{\bullet-}$ changes (at λ=420 nm). T2 was used as the absorbance of the $ABTS^{\bullet-}$ catalyst (green bar) and non-catalyst (purple bar), and the concentration of T2 was 0.6×. (green bar) H4+H5+H6+T2; (purple bar) H4+fH5+H6+T2; (orange bar) H4+H5+H6;

2.6 The principle and verification of the single-input dual-output module

Here, we implement single-input dual-output signal conversion in the DNA reaction network. To prove single-input dual-output signal conversion can be achieved, we need to prove that the single-input single-output signal conversion can be achieved separately. As shown in Figure S7, we can see that when S is added to the mixed solution of H1, H2 and H3, the fluorescence rises significantly, which indicates that the single-input single-output signal conversion has been completed.

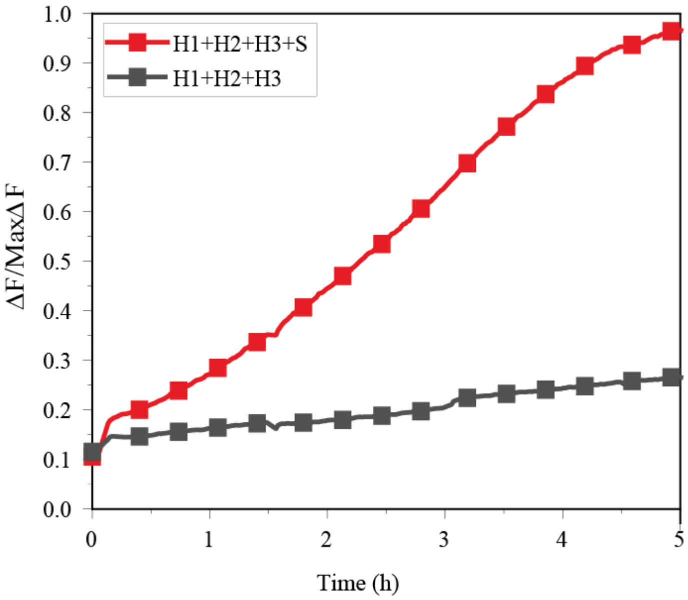

Figure S7. Time-dependent the fluorescence (at λ = 520 nm) monitoring the single-input single-output signal conversion system, in the absence (red) and presence (black) of DNA target T1.

As shown in Figure S8, we can see that when S is added to the mixed solution of H4, H5 and H6, the absorbance of the resulting $ABTS^{•-}$ rises significantly, which indicates that the single-input single-output signal conversion has been completed.

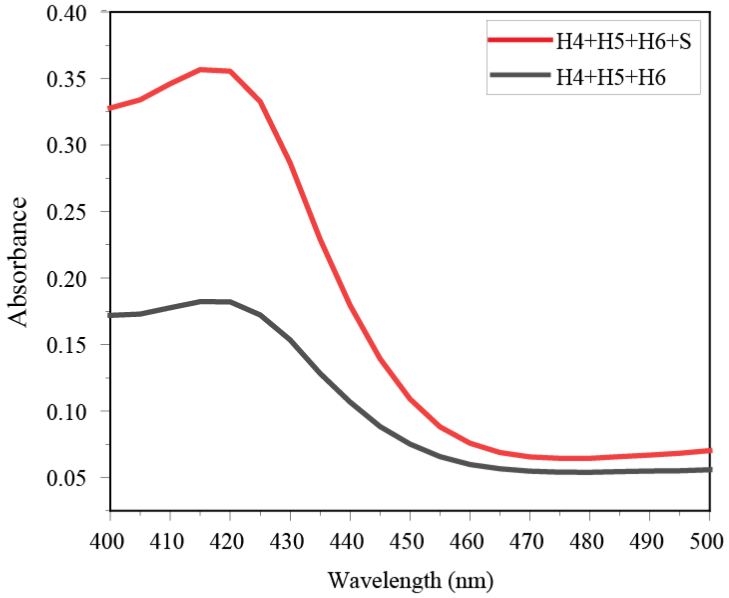

Figure S8. The absorbance of the resulting $ABTS^{•-}$ (at λ = 400 nm-500 nm) monitoring the single-input single-output signal conversion system, in the absence (red) and presence (black) of DNA target T1.

2.7 The principle and verification of the reversible regulatory DNA reaction network

Verification of the reversible regulation of the programmed DNA reaction network was conducted as follows. If we want to achieve hybrid regulation in the same network, then we need to ensure that reversible regulation can be achieved in a single network. Figure S9a shows the fluorescence changes produced by the addition of inhibitor C1 and de-inhibitor C1* at different time points (DNAzyme 1 digests the reporter strand D). First, the trigger strand was added to the reaction for 1.5 h, and the fluorescence increased significantly within 1.5 h. When C1 was added at 1.5 h, DNAzyme 1 activity was lost. The hairpin H2 with a part of the DNAzyme 1 subunit structure was replaced, and the increase of fluorescence stopped. It can be seen from Figure S9a that the fluorescence still increased at a very slow rate. The reason why the fluorescence did not stop increasing immediately is that it took time for the inhibitor C1 to completely displace H2. When the inhibitor C1 was not completely replaced, there was still a part of DNAzyme 1 that digested the reporter strand D, which produced fluorescence. When the de-inhibitor C1* was added at 3 h, the fluorescence increased again at a high rate, and the same operation was performed in three cycles. DNAzyme 2 catalyzed the oxidation of $ABTS^{2-}$ to the blue-green $ABTS^{\bullet-}$ product under the action of H_2O_2, as shown in Figure S9b. The absorbance changes of $ABTS^{\bullet-}$ at different time points with the addition of inhibitor C2 and de-inhibitor C2* are also shown. The absorbance increased significantly within 1.5 h after the triggering agent was added. When C2 was added at 1.5 h, the DNAzyme 2 was inactivated, and the hairpin H5 with a part of the DNAzyme 2 subunit structure was replaced, and the absorbance began to decline. When C2* of the disinhibition strand was added at 3 h, the absorbance of the $ABTS^{\bullet-}$ began to increase again, and the same operation was performed in three cycles. In Figures S9a and S9b, the inhibitory effect and the disinhibitory effect were both as expected.

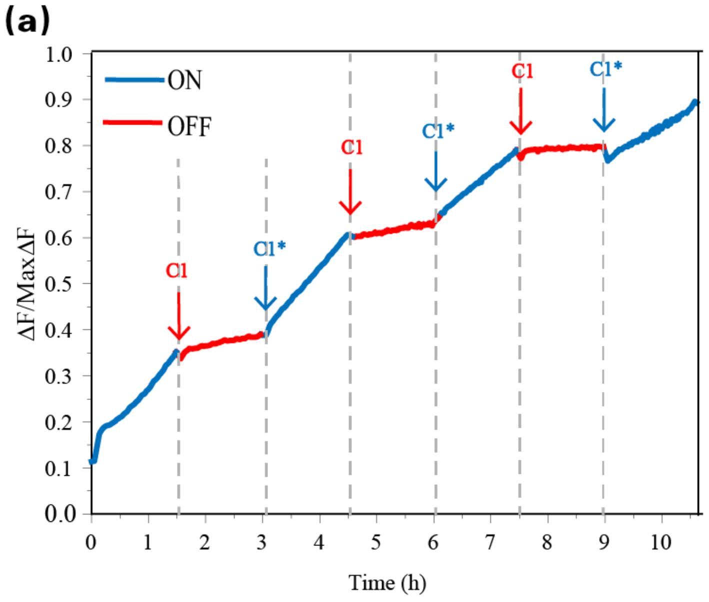

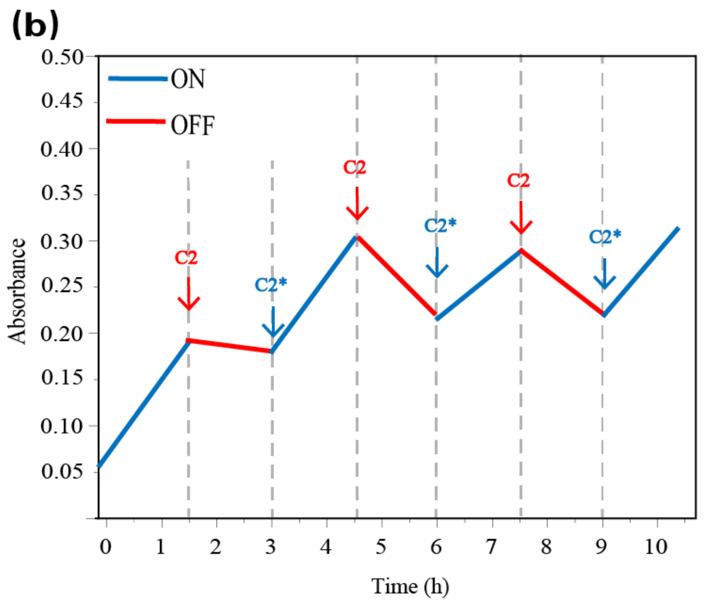

Figure S9. Time-dependent fluorescence and absorbance changes of the DNAzyme 1, and DNAzyme 2. (a) Reversible conversion between case 1 and case 2 is controlled by inhibitors C1 and de-inhibitors C1*. And inhibition and activation of DNAzyme 1 activity. (b) Reversible conversion between case 1 and case 3 is controlled by inhibitors C2 and de-inhibitors C2*. And inhibition and activation of DNAzyme 2 activity.

Table S1 DNA sequences in this study

Name	Sequences (from 5′ to 3′)	Length
**H1**	TTTTATCAGCGATCCTGACTCATACCTCACCCATGTAGGTATGAGTCAGGTCACAGTTT	59
**H2**	CCTGACTCATACCTACATGGGTGAGGTATGTTGTTACACCCATGTACCTCTC	52
**H3**	TGATATCAGCGATTAACTTAGTCAGGATCGCTGAT	35
**T1**	CTGTGACCTGACTCATACCTA	21
**D**	GAGAGGTT/rA/GGATATCA	17
**fH2**	CATGGGTGAGGTATGTTGTTACACCCATGTACCTCTCTTTTTTTTTTTTTT	52
**H4**	TTTGGGCCTGAGCCATACCTGGGCGGGAGGTATGGCTCAGGTCAGAG	47
**H5**	CCTGAGCCATACCTCCCGCCCAGGTATGATGGGTAGGGCGGG	42
**H6**	TTTGGGTGCTCAGGCCCAAA	20
**T2**	CTCTGACCTGAGCCATACCTC	21
**fH5**	CCGCCCAGGTATGATGGGTAGGGCGGGTTTTTTTTTTTTTTTTTTT	47
**S**	CTCTGACCTGAGCCATACCTCCTGTGACCTGACTCATACCT	41
**C1**	CTGTGACCTGACTCATACCTACATGGGTGATTTTTAAGTTAATCGCTTCCACGAG	55
**C1***	CTCGTGGAAGCGATTAACTTAAAAATCACCCATGTAGGTATGAGTCAGGTCACAG	55
**C2**	CTCTGACCTGAGCCATACCTCCCGCCCAGTAAATCCCAAATCTAGCAG	48
**C2***	CTGCTAGATTTGGGATTTACTGGGCGGGAGGTATGGCTCAGGTCAGAG	48
